# Methylmercury exposure and developmental neurotoxicity

**DOI:** 10.2471/BLT.14.141911

**Published:** 2014-12-19

**Authors:** Gary J Myers, Philip W Davidson, Gene E Watson, Edwin van Wijngaarden, Sally W Thurston, JJ Strain, Conrad F Shamlaye, Pascal Bovet

**Affiliations:** a University of Rochester Medical Center, 601 Elmwood Avenue Rochester, New York, NY, 14642, United States of America.; b Northern Ireland Centre for Food and Health, University of Ulster, Coleraine, Northern Ireland.; c Ministry of Health, Victoria, Seychelles.; d Institute of Social and Preventive Medicine, University of Lausanne, Lausanne, Switzerland.

We are concerned that certain aspects of the systematic review on methylmercury (MeHg) exposure from seafood consumption and the risk of developmental neurotoxicity published in the *Bulletin of World Health Organization* could be misinterpreted.^1^ Specifically, the review does not address the issue of whether naturally-occurring, background levels of prenatal exposure to MeHg from maternal fish consumption causes adverse neurodevelopmental effects in children. In our opinion, the title suggests that the article addresses this key issue, but the search terms used to review the literature and the text of the review itself clearly indicate that the focus is limited to assessing MeHg levels in infants, pregnant women, mothers and women without children. The authors base their conclusions about developmental neurotoxicity on whether or not exposures exceed the proposed permissible tolerable weekly intake (PTWI) of 1.6 μg of MeHg per kg body weight, established by the Food and Agriculture Organization (FAO) and the World Health Organization (WHO) in 2003. This PTWI was derived from risk assessment procedures using epidemiological data and is used in risk characterization. It includes a safety factor to account for uncertainty in the exposure-response data and pharmacokinetics of MeHg. Our concern is that the readers might assume that this article reviews the evidence on which reference values were based. In fact, the authors review the evidence for exposure above the reference value. The distinction is important since fish is not only the primary human source of MeHg exposure, but it is also an essential part of daily nutrition for over 2.9 billion people worldwide, most of whom reside in developing countries with limited nutritional and health resources.^2^

One of the articles identified in the systematic search was the Seychelles Child Development Study, which was cited as demonstrating “…an association of exposure *in utero* with developmental neurotoxicity…among populations that consume seafood regularly.” The study assessed prenatal exposure of MeHg and measured a median 5.9 μg/g (range: 0.5–26.7) of total mercury in maternal hair. However, it is incorrect to use this study to suggest adverse associations between prenatal MeHg exposure and children’s development, since no such association was found. The abstract of the Seychelles Child Development Study states “…no association between the maternal hair mercury level during pregnancy and an adverse neurodevelopmental outcome of the child was identified…”^3^ Indeed, twenty-five years of cross-sectional and longitudinal studies in the Seychelles where pregnant mothers consume on average over 10 fish meals per week suggests that the benefits of fish consumption significantly outweigh the possible risks of background MeHg exposure.^4^^,^^5^

The nutritional benefits of fish are considerable. Fish contain many nutrients, including lipids that are essential for human health. Lipids constitute approximately half of the human brain and a substantial part of these lipids are docosahexanoic acid (DHA). However our body can only synthesize about 5% of the DHA it needs and must take up DHA from other sources. Fish is the primary source of DHA in our diet.

Exposure to high levels of MeHg from industrial contamination in Minamata and Niigata in Japan has clearly shown that at a high level of exposure it is a neurotoxicant and that the developing fetus is most sensitive to its effects.^6^ However, there is uncertainty about the level of exposure that causes toxicity and whether evidence supports a causal link between maternal consumption of fish contaminated by current background levels of MeHg and children’s neurodevelopment. In 2010, FAO/WHO reassessed the evidence related to fish consumption and MeHg exposure. The report established guidelines for fish consumption and stated “when comparing the benefits of LCn3PUFAs [long chain n-3 polyunsaturated fatty acids] with the risks of methylmercury among women of childbearing age, maternal fish consumption lowers the risk of suboptimal neurodevelopment in their offspring compared with the offspring of women not eating fish in most circumstances evaluated.” The report recommended that member states “acknowledge fish as an important food source of energy, protein and a range of essential nutrients and fish consumption as part of the cultural traditions of many peoples” and “emphasize the net neurodevelopmental benefits to offspring of women of childbearing age who consume fish, particularly pregnant women and nursing mothers, and the neurodevelopmental risks to offspring of women of childbearing age who do not consume fish.”^7^

Policy-makers, especially in low- and middle-income countries where fish consumption is high and resources are limited, will be best served by presenting a balanced view of the evidence of the benefits and possible risks of a diet rich in ocean fish. Although we have not found evidence that the levels of MeHg exposure achieved by fish consumption in the Seychelles cause developmental neurotoxicity, we fully support the Minamata Convention on mercury regulation^8^ and encourage others to do the same. Pollution of all kinds is a public health problem and we all have a moral obligation to future generations to prevent it.

## References

[1] Sheehan MC, Burke TA, Navas-Acien A, Breysse PN, McGready J, Fox MA. Global methylmercury exposure from seafood consumption and risk of developmental neurotoxicity: a systematic review. Bull World Health Organ. 2014 4 1;92(4):254–269F. 10.2471/BLT.12.116152 24700993 PMC3967569

[2] The state of world fisheries and aquaculture. Rome: Food and Agriculture Organization of the United Nations; 2014 Available from: http://www.fao.org/3/a-i3720e/index.html [cited 2014 Oct 7].

[3] Myers GJ, Marsh DO, Davidson PW, Cox C, Shamlaye CF, Tanner M, et al. Main neurodevelopmental study of Seychellois children following in utero exposure to methylmercury from a maternal fish diet: outcome at six months. Neurotoxicology. 1995 Winter;16(4):653–64. 8714870

[4] van Wijngaarden E, Thurston SW, Myers GJ, Strain JJ, Weiss B, Zarcone T, et al. Prenatal methyl mercury exposure in relation to neurodevelopment and behavior at 19 years of age in the Seychelles Child Development Study. Neurotoxicol Teratol. 2013 Sep-Oct;39:19–25. 10.1016/j.ntt.2013.06.003 23770126 PMC3795956

[5] Strain JJ, Davidson PW, Thurston SW, Harrington D, Mulhern MS, McAfee AJ, et al. Maternal PUFA status but not prenatal methylmercury exposure is associated with children’s language functions at age five years in the Seychelles. J Nutr. 2012 11;142(11):1943–9. 10.3945/jn.112.163493 23014496 PMC3498972

[6] Harada M. Minamata disease: methylmercury poisoning in Japan caused by environmental pollution. Crit Rev Toxicol. 1995;25(1):1–24. 10.3109/10408449509089885 7734058

[7] Fisheries and aquaculture report No. 978. Rome: Food and Agriculture Organization of the United Nations; 2010 Available from: www.fao.org/docrep/014/ba0136e/ba0136e00.pdf [cited 2014 Oct 7].

[8] Minamata Convention on Mercury [Internet]. Geneva: United Nations Environment Programme; 2014. Available from: http://www.mercuryconvention.org/Convention/tabid/3426/Default.aspx [cited 2014 Nov 28].

